# 

**Published:** 1894-03-31

**Authors:** 


CONTENTS.

**Medical Protection for School Morals** - - - - - - 473
**Around the Hospitals** - - - - - - - - - - 474
**Treatment of Chloroform Col apse.** By Sir BENJAMIN WARD RICHARDSON, M.D., F.R.S. - - - - - - - 475
**On the Diagnostic Value of the Degree of Acidity of the Gastric Juice** - - - - - - - - 476
**Modern Medico-Psychology and Psychiatry** By T. S. CLOUSTON, M.D. - - - - - - - - - - - 477
**Great Achievements Under Suffering.** V.—Leopold, Duke of Albany - - - - - - - - - - - 478
**Annotations.**—Tangier as a Health Resort—Brain Scavengers—An Incompatible Mixture - - - - - - - - - 479
**Notes and News** - - - - - - - - - - 480
**Medical Progress and Hospital Clinics**—
I.—The Formation of Uric Acid and Its Effects on the System - 481
Inguinal Colotomy in Carcinoma of the Rectum - - - 482
The Treatment of Post-partum Hæmorrhage - - - - 484
MEDICAL PROGRESS—Typhoid, 485; Tetanus - - - - 486
NEW APPLIANCES AND THINGS MEDICAL - - - - - 488
**The Institutional Workshop**—
WITHIN THE HOSPITALS—Newcastle Royal Infirmary - - - 489
HOSPITAL FESTIVALS, MEETINGS, &c., 491; CONSTRUCTION NOTES 492
**Scraps and Gleanings** - - - - - - - - - 492
**The Book World of Medicine and Science** - - - - 493

EXTRA SUPPLEMENT.—THE NURSING MIRROR.

**News from the Nursing World.**—Saved, Not Wasted—Infants' Nourishment Again—Warlike Women—A High Fee—St. Bartholomew's Probationers—Testimonials—Genuine Appreciation—Leeds Ladies' Hospital Fund—Good Work at Hinckley—A New Isolation Hospital—Stirling—Queen's Nurses in Dublin—Short Items - - - - - - - ccli
**On General Nursing.** V.—Scarlet Fever (*continued*) - - ccliii
**The International Medical Congress at Rome** - - ccliv
**St. Thomas's Hospital Chapel** - - - - - - - ccliv
**Women's Work.**—Practical Massage I. - - - - - - cclv
**Notes from Melbourne** - - - - - - - - cclv
**Lectures at St. George's Hospital**—The Antiseptic System (*continued*) - - - - - - - - - cclvi
**Notes from South Africa** - - - - - - - - cclvi
**The Junius S. Morgan Benevolent Fund for Nurses** cclvii
**The Muses' Looking Glass.**—In Rural Districts—Unrecognised Perils - - - - - - - - - cclix
**Royal British Nurses' Association** - - - - - cclix

